# Influence of age and sex on physical, cardiac electrical and functional alterations in progressive hyperoxia treatment: A time course study in a murine model

**DOI:** 10.1016/j.exger.2024.112435

**Published:** 2024-04-19

**Authors:** Yashwant Ayalasomayajula, Anagha Hesaraghatta, Neha Dantuluri, Jenna Yassine, Faizan Saleem, Hussein Mansour, Chayapatou Chayawatto, Nishank Rangarajan, Sashank Rangarajan, Smrithi Krishnan, Siva Kumar Panguluri

**Affiliations:** a Department of Pharmaceutical Sciences, College of Pharmacy, University of South Florida, 12901 Bruce B. Downs Blvd., Tampa, FL 33612, USA; b Cell Biology, Microbiology and Molecular Biology, College of Arts and Sciences, University of South Florida, 12901 Bruce B. Downs Blvd., Tampa, FL 33612, USA

**Keywords:** Hyperoxia, Cardiac hypertrophy, ECG, Echocardiogram

## Abstract

Oxygen supplementation is a widely used treatment for ICU patients. However, it can lead to hyperoxia, which in turn can result in oxidative stress, cardiac remodeling, and even mortality. This paper expands upon previous research conducted by our lab to establish time-dependent cardiac changes under hyperoxia. In this study, both young and aged mice (male and female) underwent 72 h of hyperoxia exposure and were monitored at 24-hour intervals for cardiac electrophysiological and functional parameters using ECG and electrocardiogram data. Our analysis showed that young male mice experienced significant weight loss as well as significant lung edema by 48 h. Although young male mice were highly susceptible to physical changes, they were resistant to early cardiac functional and electrophysiological changes compared to the other groups. Both young and aged female and aged males developed functional impairments by 24 h of hyperoxia exposure. Furthermore, sex and age differences were noted in the onset of electrophysiological changes. While some groups could resist early cardiac remodeling, our data suggests that 72 h of hyperoxia exposure is sufficient to induce significant cardiac remodeling across all age and sex groups. Our data establishes that time-dependent cardiac changes due to oxygen supplementation can have devastating consequences even with short exposure periods. These findings can aid in developing clinical practices for individuals admitted to the ICU by elucidating the impact of aging, sex, and length of stay under mechanical ventilation to limit hyperoxia-induced cardiac remodeling.

## 1. Introduction

The use of oxygen supplementation treatments, such as mechanical ventilation, is a common practice in ICU settings to alleviate respiratory-related issues. Oftentimes, patients in these settings are faced with hypoxemia, regardless of any other diseases they may have. With hypoxemia (hypoxia), dangerously low levels of oxygen in the body results in respiratory and cardiovascular overload, which affects the hemato-logical system and results in cellular damage and organ failure (Pala Cifci et al., 2020). Thus, the goal of oxygen supplementation is to counteract the adverse effects of hypoxia in patients. It has been suggested that the use of perioperative hyperoxia can help pre-condition cardiac tissues allowing them to tolerate ischemia (Young, 2012). Furthermore, it has also been suggested that hyperoxia treatments can reduce wound infections following surgeries, as well as reduce the production of gas microemboli during cardiopulmonary bypass (Young, 2012). Hyperoxia, a condition characterized by excessive tissue oxygenation (SpO2 > 96 %), results in detrimental cardiac remodeling and functional changes (Li et al., 2007). Though most ICU clinicians acknowledge the potentially adverse effects of hyperoxia, clinicians administer higher amounts of oxygen in comparison to recommended target ranges for mechanical ventilation (Helmerhorst et al., 2014). Furthermore, a meta-analysis of 24 studies that explored the role of hyperoxia in adult patients admitted to ICUs showed that hyperoxia led to mortality in critically ill patients, especially those with extracorporeal life support (ELS) and cardiac arrest (Ni et al., 2019). Both ELS and cardiac arrest consist of unstable cardiac hemodynamics which might be attributed to impeding cardiac function via the increase of reactive oxygen species and inhibition of vasodilators such as nitric oxide (Ni et al., 2019). Hyperoxia-related mortality may also be caused by vascular resistance, which can result in reduced cardiac output, cardiac shock, and mortality (Martin et al., 2020; Ni et al., 2019; Singer et al., 2021). To add, reports suggest that the adverse effects of supplemental oxygen show sex differences (Vichare et al., 2022). Female patients have been shown to be at a greater risk for hyperoxic ICU–acquired muscle weakness (Raurell-Torreda et al., 2021). Alternatively, male patients have been shown to have a greater risk of direct lung injury due to hyperoxia in comparison to female patients, with female patients being less susceptible to oxidative stress induced by hyperoxia (Lingappan et al., 2013; Macak-Safranko et al., 2011).

Our lab pioneered hyperoxia research by analyzing the impact of age, sex, and comorbid conditions on hyperoxia-induced cardiac pathophysiology in mouse models, suggesting cardiac remodeling and cardiac injury to be a result of hyperoxia (Panguluri et al., 2013; Rodgers et al., 2019a; Rodgers et al., 2021; Saleem et al., 2023; Vichare et al., 2022). We observed that 72 h of hyperoxia treatment resulted in left ventricular hypertrophy, arrhythmias, and prolonged JT and QTc intervals, indicating detrimental electrical, structural, and functional cardiac remodeling (Panguluri et al., 2013). Following this research, our lab observed hyperoxia-induced arrhythmias and time-dependent cardiac dysfunction in adult C57BL/6 J male mice as early as 24 h in hyperoxic conditions (Rodgers et al., 2021). Specifically, cardiac electrical remodeling preceded structural and functional cardiac remodeling with all observed cardiac parameters worsening in increased exposure to hyperoxia (Rodgers et al., 2021). We also reported that sex differences are present with 72 h of hyperoxia treatment, in which young female mice showed higher mortality and cardiac changes than the age matched male group (Rodgers et al., 2019b). Our lab also discussed age-dependent cardiac effects in mice indicating that aged mice are at higher risk under hyperoxia compared to their younger counterparts (Vichare et al., 2022).

Our previous time-course experiment outlined the time-dependent effects of hyperoxia in young male mice (Rodgers et al., 2019b). In this manuscript, we further investigated time-dependent changes in hyperoxia-induced cardiovascular pathophysiology in both male and female mice from both young and aged groups. The data presented in this study will help us to understand the influence of age and sex on hyperoxia-induced adverse events in a time-dependent manner. The data obtained from this study can be further validated in clinical and translational research, helping us to improve patients’ outcomes in critical/emergency care and ICU settings.

## 2. Materials and methods

### 2.1. Animals

In this study, C57BL strain mice (stock#000664 from Jackson Laboratories) of both male and female sex were utilized. The mice were then divided into young (~8–10 weeks of age) and aged (~72 weeks of age) groups. Each mouse was given access to water and food ad libitum, and they were maintained on a 12 h light/dark cycle. There were *n* = 20–30 mice per experimental group. The number of mice in each group was determined by a power analysis calculator (http://www.biomath.info/power/prt.htm). This ensured that the experimental group size was proportionate to the sample size. The raw data for the young male mice was retrieved from our past investigation.

### 2.2. Hyperoxia exposure

Before each of the treatment periods, all the mice were weighed and numerically labeled. Each of the experimental groups were subjected to either hyperoxia or normoxia exposure as published previously (Panguluri et al., 2013). The mice in the hyperoxia group were exposed to >90 % oxygen in a 50×50×30 cm airtight chamber, using the study protocols previously used in our lab (Panguluri et al., 2013). Oxygen levels were monitored to be >90 % using an oxygen analyzer (Vascular Technology, Chelmsford, MA). Following the treatment periods (24 h, 48 h, and 72 h), the mice were weighed followed by 2D-echocardiogram and ECG data collection as published in our previous studies (Vichare et al., 2022). The mice were then euthanized using 50 mg/kg euthasol through intraperitoneal injection. Following euthanization, the mice’s hearts, lungs, and blood were collected. Plasma serum was collected from the blood through a centrifuge, by spinning at 5500 rpm for 5 min immediately following collection. Hearts, lungs, and plasma were stored at −80C until further analysis.

### 2.3. Physical parameters

Body weights were collected prior to normoxia or hyperoxia treatment periods (24 h, 48 h, and 72 h) and normalized with tibia length. Lung wet and dry weights were used to calculate lung edema using wet/dry ratios as described in our previous publication (Rodgers et al., 2021).

### 2.4. Echocardiogram

The Vevo 3100LT Ultrasonograph (VisualSonics) equipped with a 30 MHz transducer was used to perform transthoracic 2D-echocardiograms. The echocardiograms of mice exposed to either normoxia or hyperoxia were performed using the procedure described previously (Rodgers et al., 2021). Mice were anesthetized using 1 %–1.5 % isoflurane for immobilization during echocardiograph collection. A 2-dimensional parasternal short-axis view and parasternal long-axis view of the midpapillary muscle were imaged, followed by 30-s recordings across the anterior wall and posterior wall using the M-mode in the parasternal short-axis view. The VevoLab Software was used to measure left ventricular anterior wall (LVAW), systolic and diastolic left ventricular interior diameter (LVID) and left ventricular posterior wall thickness (LVPW). Ejection Fraction (EF%) was determined using the End Diastolic Volume (EDV) and End Systolic Volume (ESV) with the following formula: (EDV – ESV)/EDV × 100 %, Fractional Shortening (FS%) was determined using the Left ventricular internal diameter end diastole (LVID;d) and Left ventricular internal diameter end systole (LVID;s) with the following formula: (LVID;d – LVID;s)/ LVID;d × 100 %. Ejection Fraction (EF%) was determined using (EDV – ESV)/EDV × 100 %, Fractional Shortening (FS%) was determined using (LVID;d – LVID;s)/ LVID;d × 100 %, Stroke Volume (SV) was determined using (EDV – ESV), and Cardiac Output (CO) was determined using (heart rate x SV).

### 2.5. Electrocardiogram (ECG)

Following anesthetization using 1 %–1.5 % isoflurane, surface probes in lead II configuration were inserted into the mice, using the same procedure as described previously (Panguluri et al., 2013). The ECGs were recorded for 30-second intervals, followed by ECG analysis using LabChart 7.2 software (AD Instruments). RR, PR, QRS, JT, and QT intervals were measured in a similar manner as described in a previous publication (Chapalamadugu et al., 2015). Bazett’s formula, QTc = QT/RR1/2, was used to determine corrected heart rate intervals (QTc).

### 2.6. Further analysis

Following the treatment periods and collection of ECG and echocardiogram data, the mice were then euthanized using 50 mg/kg euthasol through intraperitoneal injection. Following euthanization, the mice’s hearts, lungs, and blood were collected. Plasma serum was collected from the blood through a centrifuge, by spinning at 5500 rpm for 5 min immediately following collection. Hearts, lungs, blood, and plasma were stored at −80C until further analysis. Lung wet and dry weights were used to calculate lung edema using wet/dry ratios using the same methods described in our previous publication (Rodgers et al., 2021).

### 2.7. Statistical analysis

A multi-way ANOVA test was used to calculate differences between groups as they were influenced by treatment, age, and sex. The Turkey Honest significant difference (Tukey HSD) was used to determine pair-wise multiple comparisons. All tests were run through the R statistical program, along with Pearson’s correlation matrix of outcomes using the corrplot package.

## 3. Results

### 3.1. Physical parameters

**Fig. 1.**
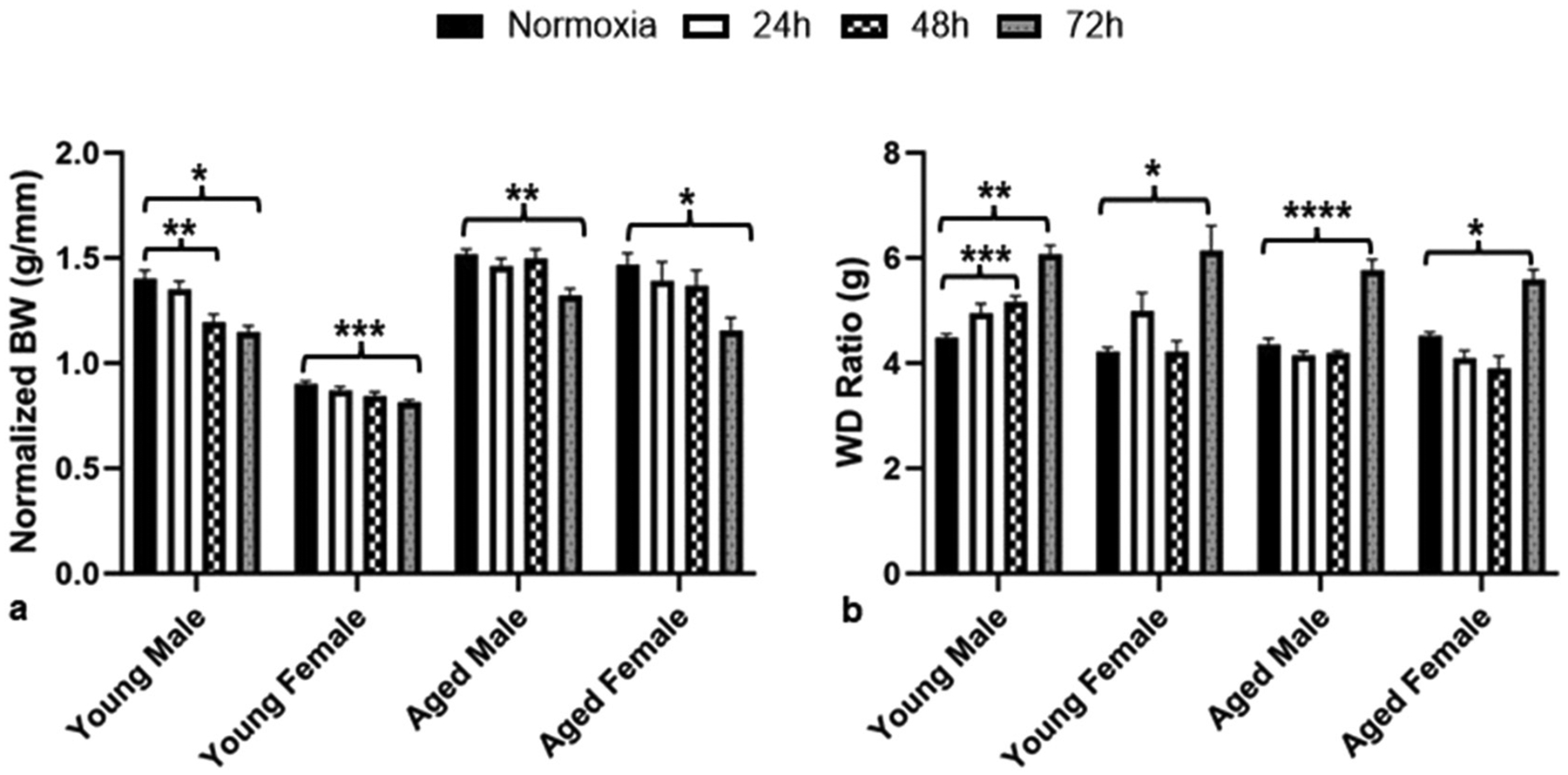
Hyperoxia-induced changes in physical parameters are not significant until 72 h. (a) Body weight (BW) for all experimental groups normalized to tibia length (g/cm), (b) Lung wet/dry (WD) ratio for all experimental groups. For all data, error bars represent ± SEM. *p < 0.05, **p < 0.005, ***p < 0.0005. * Represents *p*-value between hyperoxia and normoxia of same sex and age.

We previously observed significant changes in physical parameters such as body weight and lung edema after 72 h of hyperoxia exposure regardless of age and sex (Panguluri et al., 2013; Rodgers et al., 2021; Vichare et al., 2022). Therefore, this study also evaluated changes in body weights and lung edema after each time-point of hyperoxia or normoxia treatment. As reported earlier (Vichare et al., 2022), older mice were heavier than young mice regardless of exposure conditions and sex (Fig. 1a). Our results showcased a significant decrease in body weight (normalized to tibia length) in 72 h hyperoxia-treated mice compared to normal air controls in all ages and sexes (Fig. 1a). Only the young male mice group showed a significant decrease in body weights compared to their normoxia controls as early as 48 h, whereas all other groups did not show any significant change in body weights until 72 h of hyperoxia (Fig. 1a).

Lung edema was examined by measuring lung wet-to-dry weight ratios. Similar to our previous reports (Panguluri et al., 2013; Rodgers et al., 2021; Vichare et al., 2022), significant lung edema was observed in all groups at 72 h hyperoxia (Fig. 1b).

### 3.2. Functional parameters

**Fig. 2.**
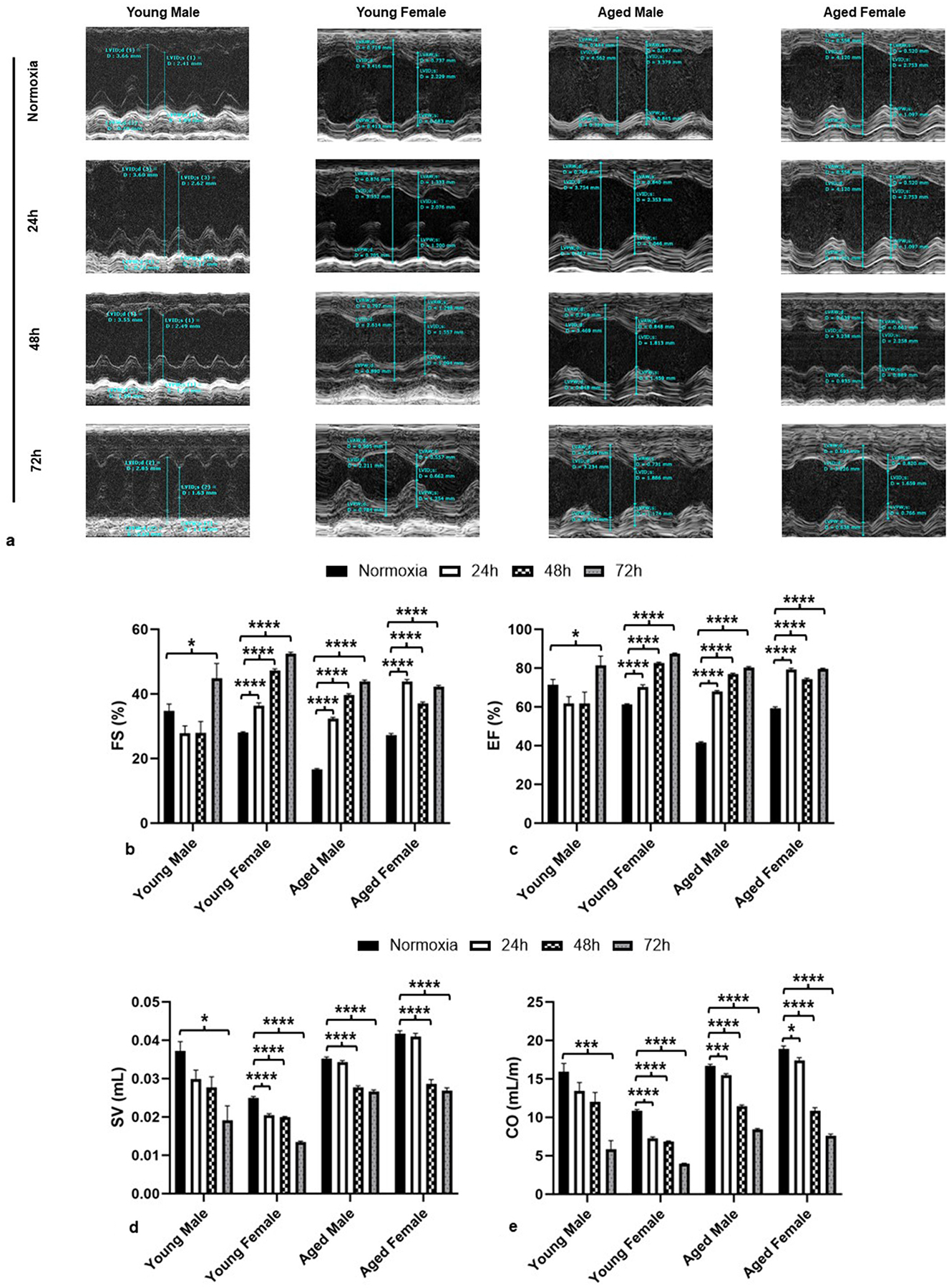
Hyperoxia significantly changed cardiac functioning as early as 24 h in all groups except young males. (a) M-mode parasternal short axis view in two-dimensional echocardiography for normal air and hyperoxia groups (young and aged; male and female), (b) percent fractional shortening (%FS), (c) percent ejection fraction (%EF), (d) stroke volume (SV), and (e) cardiac output (CO) in hyperoxia/normoxia treated mice. For all data, error bars represent ± SEM. *p < 0.05, **p < 0.005 ***p < 0.0005. * Represents p-value between hyperoxia and normoxia of same sex and age.

We analyzed cardiac function via 2-D echocardiography following exposure to either normoxia or hyperoxia (24 h, 48 h, and 72 h). The parasternal short-axis view was recorded across the anterior and posterior walls with the 2-D guided M-mode (Fig. 2a). Ejection Fraction (%EF), Fractional Shortening (%FS), Stroke Volume (SV), and Cardiac Output (CO) were calculated.

Our data showed that hyperoxia significantly increased %FS and % EF at 72 h hyperoxia across all groups (Fig. 2b & c). Except for the young male mice group, all other groups displayed a significant increase in %FS and %EF as early as 24 h, which consistently increased after 48 h or 72 h (Fig. 2b & c), while the young male group showed no significant change at 24 h or 48 h but showed a significant increase at 72 h.

Additionally, we also observed a significant decrease in Stroke Volume (SV) and Cardiac Output (CO) at 72 h of hyperoxia across all age and sex groups (Fig. 2d & e). Other than the young male mice group, all other groups showed a significant decrease in SV as early as 48 h (Fig. 2d), whereas CO decreases were observed as early as 24 h (Fig. 2e). The decline in SV and CO was more acute in young mice compared to aged mice, suggesting the susceptibility of the young group to cardiac dysfunction under prolonged hyperoxia exposures compared to their aged counterparts (Fig. 2d & e). Additionally, the reduction in SV and CO was more prominent in females than in males in both age groups (Fig. 2d & e).

### 3.3. Electrophysiological parameters

**Fig. 3.**
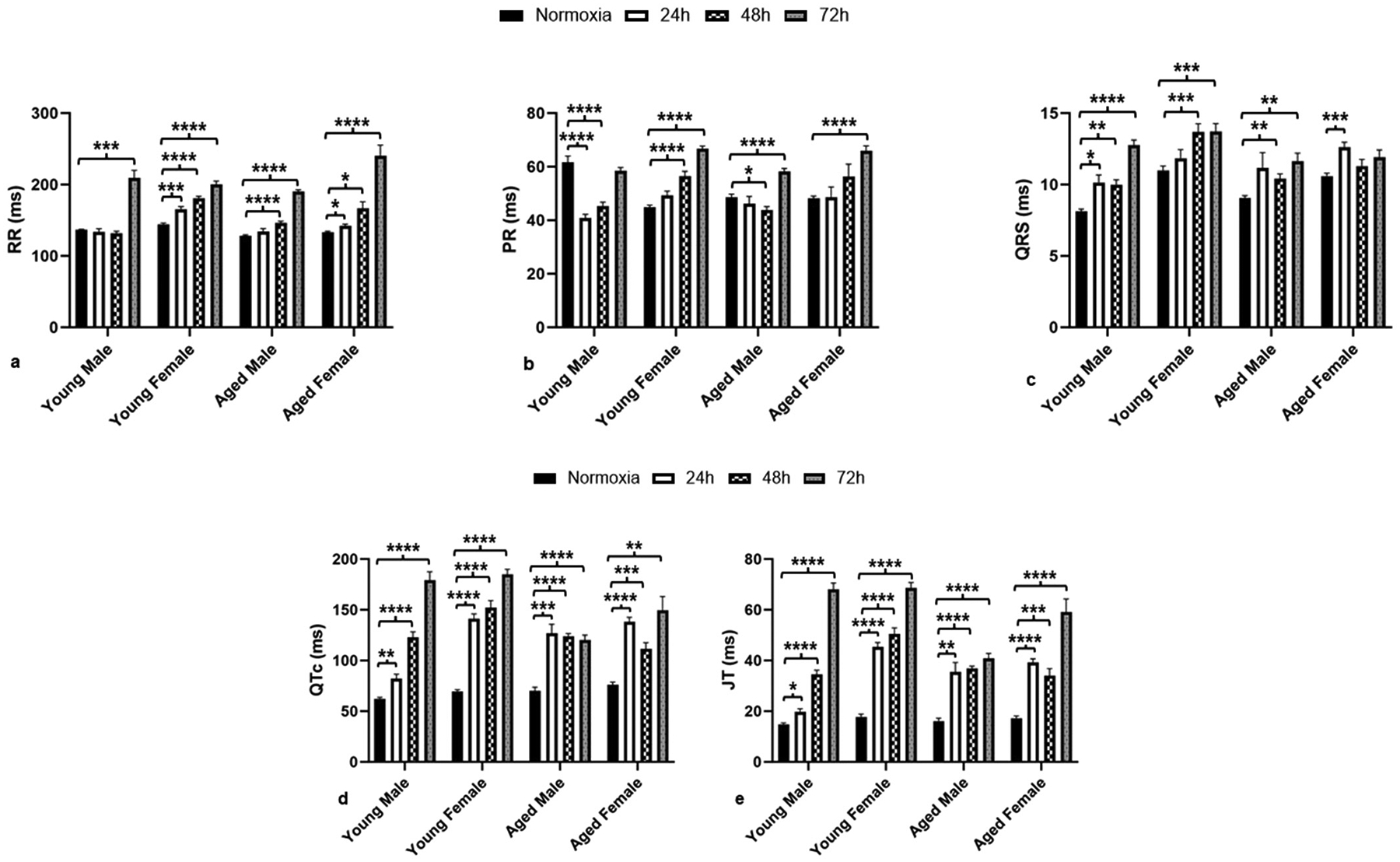
Hyperoxia-induced cardiac electric remodeling is significant as early as 24 h in all groups. (a) RR interval, (b) PR interval, (c) QRS interval, (d) QTc interval, (e) JT interval. All these intervals were recorded and reported in milliseconds (ms). Error bars represent ± SEM. *p < 0.05, **p < 0.005 ***p < 0.0005. * Represents p-value between hyperoxia and normoxia of same sex and age.

Previous research has shown that mice develop electrical disturbances (arrhythmias, QTc and JT prolongation) as early as 24 h in young male groups under hyperoxia (Chapalamadugu et al., 2015; Panguluri et al., 2013; Rodgers et al., 2018; Rodgers et al., 2021). In this study, we also observed a significant increase in QTc and JT intervals in all groups regardless of age and sex as early as 24 h of hyperoxia exposure (Fig. 3d & e). Except for the young male group, all groups showed a significant increase in RR interval as early as 24 h (both young and aged female) or 48 h (aged male), which consistently increased with the length of hyperoxia exposure (Fig. 3a). While the young male group showed a significant decrease in PR interval as early as 24 h and reverted back to normoxia levels after 72 h of hyperoxia (Fig. 3b), all other groups showed a significant increase in PR intervals with hyperoxia exposure as soon as 48 h or at least by 72 h. It was also observed that the aged female group showed a significant increase in QRS interval as soon as 24 h of hyperoxia but lost its significance after 48 h (Fig. 3c), whereas all other groups showed significantly increased QRS intervals consistently with hyperoxia exposure.

Comparing between sexes, the female groups showed significantly higher RR, PR, QRS, QTc, and JT intervals in both ages, indicating the susceptibility of females to electrophysiological changes under hyperoxia. Comparing between ages, aged females showed significantly higher RR intervals compared to young female groups (Fig. 3a), whereas the young group in both males and females showed significantly higher QTc and JT intervals at 72 h of hyperoxia compared to their aged counterparts (Fig. 3d & e).

## 4. Discussion

Hyperoxia is a key component of the treatment for circulatory shock, described as resulting from a deficit of oxygen, or the imbalance between oxygen supply and demand which must be corrected for survival, is often seen in patients in critical care units (Hafner et al., 2015). Oxygen is a vital component of various cellular processes, including adenosine triphosphate (ATP) synthesis; however, it also has strong oxidizing qualities in the form of reactive oxygen species, that can harm many biological molecules (Hafner et al., 2015). As such, the use of hyperoxia for treatment can have detrimental consequences. Often, mechanical ventilation is used to administer hyperoxia treatments; as of 2023, the relative increase from the previous year in the use of mechanical ventilators for adults was 31.5 %, especially as a result of the COVID-19 pandemic (Tsai et al., 2022). Our lab has investigated the consequences of hyperoxia for a decade, focusing on the cardiac remodeling and physical changes observed in various mice models. Initially, we reported that young male mice undergo left ventricular hypertrophy following 72 h hyperoxia exposure; furthermore, ion channel remodeling and changes in transcriptional factor regulation were observed (Panguluri et al., 2013). Furthermore, our investigation of potassium currents in male mice showed that prolonged outward potassium currents, shorter action potentials, and increased transcription and protein levels of Kv1.5 and KChIP2 following 72 h hyperoxia (Vysotskaya et al., 2018). These findings were further supported in a later experiment, where we reported that male mice developed arrhythmias, cardiac dysfunction, and ion channel remodeling in a time-dependent manner (Rodgers et al., 2021). As age and sex are also significant variables affecting response to hyperoxia, we also investigated how sex and age play a role in cardiac remodeling both independently, as well as following hyperoxia exposure. We noted that female mice were highly susceptible to lower heart rates, elevated RR intervals, and higher mortality rates after hyperoxia treatment (Rodgers et al., 2018). Coupled together, we noted that sex and aging both play a role in the risk for CVD, along with other risk factors such as obesity and diabetes (Rodgers et al., 2019b). We also noted that reduced hormone levels may be a reason for the development of CVD in older populations; however, hormone replacement therapies were not an effective treatment for reducing this risk (Rodgers et al., 2019b). Additionally our previous experiments also suggest that while sex and age differences exist, hyperoxia was the main factor that affected most cardiac parameters, which was followed by age, and finally sex (Vichare et al., 2022). To investigate the influence of age and sex on hyperoxia-induced cardiac remodeling in a time-dependent manner further, we designed this time course experiment with aged and young mice of both sexes that were subjected to hyperoxia or normoxia for various time periods of exposure.

### 4.1. Physical and cardiac changes are sex-dependent and worsen with increased hyperoxia exposure

#### 4.1.1. Young males are more susceptible to physical changes at early hyperoxia exposure treatment periods

Young male mice showed a significant decrease in body weight compared to normoxia as early as 48 h of hyperoxia exposure, whereas all other groups showed significant weight loss at 72 h (Fig. 1a). Our findings are consistent with previous findings which showed that hyperoxia resulted in decreased body weights (Bojkovic et al., 2021; Rodgers et al., 2018; Rodgers et al., 2021; Vichare et al., 2022).

Hyperoxia-dependent weight loss was reported in previous studies as well (Barazzone-Argiroffo et al., 2001; Coursin et al., 1987). In another study investigating the relationship between hyperoxia, leptin, and weight loss, it was found that weight loss occurred independent of leptin levels, indicating that weight loss resulted from hyperoxia exposure (Barazzone-Argiroffo et al., 2001). However, the precise reason behind the weight loss observed in our findings has not been extensively investigated. Clinically, individuals on mechanical ventilation often experience weight loss due to inadequate nutrition, muscle atrophy, and a decline in lean body mass, which can vary depending on the duration of their intensive care unit stay (Seo et al., 2011). We also reported that fluid intake under hyperoxic conditions significantly reduced body weights but not electrical parameters (Rodgers et al., 2021). Additionally, significant lung edema was observed as early as 48 h in young males (Fig. 1b). Similar studies have also noted the increased susceptibility in male mice to lung injury as compared to female mice (Lingappan et al., 2013; Lingappan et al., 2015). This phenomenon, described as Hyperoxic Acute Lung Injury or HALI, is known to be a result of prolonged breathing of F_I_O_2_ > 0.9 (Kallet and Matthay, 2013; Mizushina et al., 2015). Due to high levels of oxygen from hyperoxia exposure, a large amount of reactive oxygen species is produced (Bhandari, 2008; Kallet and Matthay, 2013; Mach et al., 2011), which cannot be controlled by the body’s natural anti-oxidizing properties, resulting in various cellular structures becoming damaged (Kallet and Matthay, 2013). This could be linked to the increased susceptibility of the epithelial wall in the lungs to inflammatory responses, damaging the alveolar capillaries, resulting in lung edema and reduced gas exchange (Mach et al., 2011). Furthermore, another study on HALI observed that hyperoxia which results in oxidative stress can activate necroptosis, or a regulatory inflammatory form of cellular death, which may be a potential pathway by which HALI results (Han et al., 2018). Taken together, our data suggests that young males are at the highest risk for physical parameters.

#### 4.1.2. Females and older males are more susceptible to cardiac functional impairments at early hyperoxia treatment periods

Aged males and both young and aged females showed significant changes in FS% (Fig. 2b), EF% (Fig. 2c), and CO (Fig. 2e) as early as 24 h. Previous studies have noted that the phenomenon by which ejection fraction is >70 % is known as hyperdynamic left ventricle ejection fraction (HDLVEF) (Rahman et al., 2022). Our findings were further supported by a study of ICU patients which found that HDLVEF was associated with the female sex and increased age (Paonessa et al., 2015). While it has previously been reported (Paonessa et al., 2015) that HDLVEF was associated with the female sex in critically ill patients, our study shows that aged males are also susceptible to this risk. Another study on ICU patients specifically noted that the combination of the male gender and aging population, or the aging male population, were highly susceptible to HDLVEF (Ma et al., 2022), further corroborating our finding that aging males are highly susceptible to these changes in cardiac function parameters. Various studies have also noted that HDLVEF is associated with increased mortality in COVID-19 patients (Rahman et al., 2022). The presence of HDLVEF in all female hyperoxic mice and aged male hyperoxic mice as early as 24 h could potentially be linked with an increased risk of 28-day mortality, hypertension, and HCM in these groups, whereas 72 h of hyperoxia is sufficient to cause such effects in young males (Aune et al., 2021). As noted above, all groups except young males demonstrate HDLVEF as early as 24 h (Fig. 2c), suggesting young male mice do not show functional abnormalities until later stages of hyperoxia treatment compared to all other groups. Furthermore, decreases in cardiac output were demonstrated as early as 24 h in all the groups, except young males, as observed in Fig. 2e. This could be linked to a reduced heart rate as early as 24 h in all other groups except young males, which only demonstrate a significant change after 72 h of hyperoxia exposure (Fig. 3a). Sex differences were observed as well, with female mice demonstrating a significant reduction in SV and CO compared to males (Fig. 2d and e). While both young and aged females demonstrate a significant decline in CO by 24 h, the CO decline in young females at 24 h is at a higher magnitude, suggesting that young females are highly susceptible to the negative functional consequences of hyperoxia. This finding was previously reported by our lab, where we noted that while all groups experienced reductions in SV and CO, the young female group showed a more significant decrease than any of the other groups (Vichare et al., 2022). The high mortality reported in young female mice after 72 h of hyperoxia in our previous studies (Rodgers et al., 2019b) may be due to the extremely low CO in these groups compared to all other groups. Other clinical studies also reported decreases in SV (Busani et al., 2021), along with reductions in CO (Brugniaux et al., 2018; Busani et al., 2021; Smit et al., 2018), which supports our findings of hyperoxia-induced decreases in SV and CO. As such, our findings suggest that female groups are at higher risk of change in functional parameters than the male groups.

#### 4.1.3. Females demonstrate an earlier onset of negative electrophysiological implications

While females demonstrate an increased RR interval as early as 24 h, males do not show any significance until 48 h (Fig. 3a). The RR interval, which measures the time between consecutive R waves, is used to determine the regularity of heart rates. As observed in our previous papers, females demonstrate a significantly longer RR interval, or bradycardia, compared to their male counterparts (Rodgers et al., 2019b; Vichare et al., 2022). While the JT and QTc intervals increased steadily in both aged groups and the young males, young females demonstrated an over twofold increase as early as 24 h (Fig. 3d and e). This increase in JT and QTc following hyperoxia was reported in our previous study as well (Chapalamadugu et al., 2015). The JT interval is used to determine partial repolarization, and the QTc interval is a measure of ventricular repolarization, which has been corrected for heart rate. Increased JT and QTc intervals suggest repolarization abnormalities, both of which are considered the strongest risk factors for mortality (Zulqarnain et al., 2015). The PR interval, which measures the time of the P wave to indicate atrial depolarization. Whereas the QRS complex indicates ventricular depolarization, which is used to determine the regularity of conduction to the AV node. PR elongation was suggested to be sufficient to induce atrial fibrillation (AF), indicating abnormal heart rhythms (Bidstrup et al., 2013). PR elongation has also been linked to ischemic strokes, heart attacks, and cardiovascular death (Chan et al., 2017). PR elongation was observed in young females as early as 48 h (Fig. 3b), which suggests that this group can develop AF as early as 48 h of hyperoxia exposure (Carlsson et al., 2010; Schmidt et al., 2011). Therefore, while all groups are susceptible to AF and other arrhythmias via electrical remodeling, young females are most susceptible and can show the phenotype even at shorter periods of hyperoxia exposure. Patients with AF have been shown to have a 4 times greater rate of mortality than the general population (Lee et al., 2018). This PR elongation at an early stage of hyperoxia may be linked to the high mortality rate reported in young female mice in our previous report (Rodgers et al., 2019b). Taken together, our analysis suggests that young females are the most susceptible to cardiac electrical remodeling, followed by aged females, aged males, and young males.

## 5. Conclusion

While mechanical ventilation, using hyperoxia, is common in critical care units, there are various physical as well as cardiac-related risks associated with this method. In our previous reports, we examined how hyperoxia results in changes in cardiac physiology. Furthermore, gender and age are independent risk factors in these observed changes in cardiac structure and function. In this study, we focus on identifying the sequence of cardiac events under hyperoxia exposure in a time-dependent manner, as we know that length of stay is associated with mortality in ICU patients. Our findings suggest that young males are highly susceptible to physical changes at earlier time periods. On the other hand, females and aged males are highly susceptible to cardiac functional impairments at earlier hyperoxia exposure treatment periods, while both aged and young female groups were observed to develop negative cardiac electrophysiological outcomes at early time periods. While physical parameters are more affected in young males, both female groups exhibit a higher susceptibility to cardiac functional and electrophysiological abnormalities. While research has suggested that prolonged breathing of SpO2 > 98 % can result in negative functional, structural, and electrophysiological parameters, our research suggests the consequences of hyperoxia may begin at much earlier intervals. Significant weight loss, increase in EF% and FS%, decrease in SV and CO, and prolonged QTc, RR, and JT intervals were observed within their respective groups as early as 24 h of hyperoxia exposure. Taken together, our research suggests that these time-dependent physical and cardiac changes support our claim that oxygen supplementation treatments can have devastating consequences even at short exposure periods depending on the age and sex of the patients admitted to ICU or critical care units.

## Data Availability

Data will be made available on request.
